# Fueling a Hot Debate on the Application of TiO_2_ Nanoparticles in Sunscreen

**DOI:** 10.3390/ma12142317

**Published:** 2019-07-20

**Authors:** Shweta Sharma, Rohit K. Sharma, Kavita Gaur, José F. Cátala Torres, Sergio A. Loza-Rosas, Anamaris Torres, Manoj Saxena, Mara Julin, Arthur D. Tinoco

**Affiliations:** 1 Department of Environmental Sciences, University of Puerto Rico Río Piedras, 17 AVE Universidad STE 1701, San Juan, PR 00925-2537, USA; 2 Department of Chemistry, University of Puerto Rico Río Piedras, 17 AVE Universidad STE 1701, San Juan, PR 00925-2537, USA; 3 Biochemistry & Pharmacology Department, San Juan Bautista School of Medicine, Caguas, PR 00726, USA; 4 Department of Chemistry, Syracuse University, Syracuse, NY 13244, USA

**Keywords:** titanium dioxide, nanoparticles, solubility, toxicity, skin, safety

## Abstract

Titanium is one of the most abundant elements in the earth’s crust and while there are many examples of its bioactive properties and use by living organisms, there are few studies that have probed its biochemical reactivity in physiological environments. In the cosmetic industry, TiO_2_ nanoparticles are widely used. They are often incorporated in sunscreens as inorganic physical sun blockers, taking advantage of their semiconducting property, which facilitates absorbing ultraviolet (UV) radiation. Sunscreens are formulated to protect human skin from the redox activity of the TiO_2_ nanoparticles (NPs) and are mass-marketed as safe for people and the environment. By closely examining the biological use of TiO_2_ and the influence of biomolecules on its stability and solubility, we reassess the reactivity of the material in the presence and absence of UV energy. We also consider the alarming impact that TiO_2_ NP seepage into bodies of water can cause to the environment and aquatic life, and the effect that it can have on human skin and health, in general, especially if it penetrates into the human body and the bloodstream.

## 1. Introduction

Titanium is the ninth most abundant element in the earth’s crust and is widely recognized for its strength, long-term endurance, and electronic properties, and for these reasons it is incorporated in many different materials [1]. The value of the metal has transcended to its successful use by humans for dental and orthopedic prosthetics [2,3] and in sunscreens as titanium dioxide (TiO_2_) [4]. The metal, however, remains largely unappreciated for its biological importance despite many examples of its benefit to certain plants [5,6,7] and animals [8,9,10]. Even within the human body, there is strong evidence for a biological function—a structural templating role. Titanium features the property of osseointegration, a pioneering and serendipitous discovery made by Dr. Per-Ingvar Brånemark in the 1950s [11,12]. That is, the metal is able to integrate and be structurally accepted by bone without the requirement of soft tissue connection. For this reason, it is widely used in alloy form in different prosthetics. It essentially aids in the healing and regrowth of bones, and in many applications, substitutes for bones. In the context of titanium-containing prosthetics, osseointegration may be the result of the surface of the implant forming a layer of titanium oxide. This layer protects the implants from corrosion (an excellent feature for structural integrity) and favorably interacts with biomolecules of the body [13]. The surface becomes highly protein-covered due to strong protein affinity to the titanium oxide, a demonstration of excellent biocompatibility, and serves as a biomineralization template [14]. 

Human application of titanium in materials and skin products has long been driven by the belief that it is safe and is inert to biochemical reactivity. Recently though, increased levels and/or biotransformation of the metal within the human body has captured people’s interest. The body’s interaction with titanium-containing prosthetics can extend beyond a simple passive, biocompatible one. The metal from these materials demonstrates surprising reactivity in biological fluids, is able to be released and enter into the bloodstream in titanium (IV) (Ti(IV)) ion soluble form, and as TiO_2_, is notoriously insoluble [15]. Older beliefs regarding titanium’s inertness are likely due to overly simplistic stability studies of the metal (in pure or alloy form) performed in water at physiological pH values. Such solutions do not properly represent the diverse constituent of species in biological fluids and, thus, the contribution that biomolecules play in metal speciation in the body [16,17,18]. Biomolecules bind to titanium on implant surfaces or fragments dispersed due to wearing and can lead to its dissolution [19] and transportation throughout the body. That titanium leaches from implants and is present at significantly elevated levels in the blood of people with such implants [15] has led to growing concerns about the long-term stability of these products and their impediments to human health. Some of the potential problems reported are that the titanium can corrode and lead to implant breakage [20], can generate reactive oxygen species following release from implants [21], and can produce a type IV allergy toward the metal (rare) [22,23]. Severe health issues because of metal leaching from prosthetics have been reported particularly for cobalt, which has led to the formal medical term arthroprosthetic cobaltism [24,25,26,27,28]. Toxicological problems due to the leached soluble Ti(IV) ion form of the metal has been the subject of an extensive review by Piekoszewski et al. [29]. Another study determined the concentration of “leached” titanium in either soluble (using Ti(IV) tricitrate as an appropriate blood small-molecule model) or TiO_2_ nanoparticle formulation that can lead to toxicity [19]. At concentrations ≥10 μg/mL, both formulations led to the significant antiproliferation of MC3T3 murine osteoblasts and human colorectal adenocarcinoma cell line HT29 [19]. Also at these levels (~200 μM), soluble Ti(IV) demonstrated cytotoxicity [19] most likely due to Ti(IV) binding and fragmentation of DNA [30]. Such concentrations largely exceed the concentrations of Ti typically found in the blood of people with Ti-containing implants (≤0.25 μM) [19], which suggests that toxicological concerns over leached elevated Ti levels may not be warranted except for possible localized high concentrations. Furthermore, we have recently proposed that citrate and the iron-transport protein serum transferrin may work in synergism in blood to regulate Ti(IV) ion uptake into cells and protect them from the cytotoxic properties of the metal [31,32,33].

**Figure 1:**
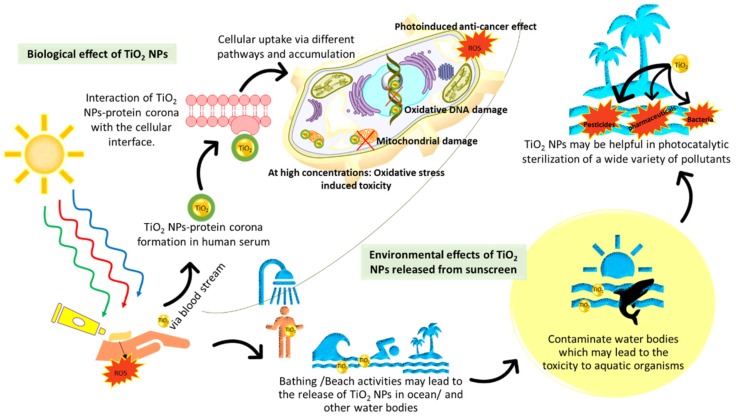
Environmental and biological effects of the application of TiO_2_ nanoparticles (NPs) in sunscreen. The NPs can pollute water bodies and possibly hurt aquatic life, but also serve a beneficial photocatalytic sterilization function. In humans, the NPs may translocate into the body. There is evidence for proteins forming a protein corona around the NPs and influencing their cellular uptake. There are several UV and non-UV debilitating cellular effects caused by TiO_2_ NPs. In both water bodies and humans, NP solubilization can occur, which produces Ti(IV) ions (not depicted) and effects similar to the NPs.

An area where toxicological concerns regarding human application of Ti has not been as well explored is the use of TiO_2_ in sunscreen. The function of sunscreen is to protect skin from harmful ultraviolet A (320–400 nm) and ultraviolet B (290–329 nm) radiation, which can cause mutations and metabolic effects [34,35]. UVB is particularly dangerous in long-term exposure because it is directly absorbed by DNA, giving rise to dimeric photoproducts between adjacent pyrimidine bases [36]. Active ingredients in sunscreen come in two forms, inorganic (mineral/physical blocker) and organic (chemical) filters. Inorganic filters like TiO_2_ nanoparticles (NPs) display both light scattering (high refractive indices) and UV absorption properties [4]. In contrast, chemical filters in the form of organic compounds solely absorb UV radiation [37]. TiO_2_ nanoparticles are widely used in sunscreen because of their excellent semiconducting properties, ease of processing, and the long-held belief that the material is biologically inert. Nonetheless, new considerations must be made regarding TiO_2_ bioactivity especially in light of its seepage into bodies of water and the different routes by which it may enter the human body. This review will explore the potentially alarming impact that TiO_2_ can have on aquatic life and on human health by evaluating its physicochemical and biochemical properties and identifying the molecular mechanisms that can affect its stability, solubility, and reactivity in living organisms (Figure 1).

## 2. Sunscreen Exploits the Semiconducting Property of TiO_2_

In sunscreen, TiO_2_ can exist in conventional (amorphous) or nanoparticle forms. The conventional form creates a milky white appearance that, while effective for UV scattering, can be aesthetically unpleasing. The nanoparticle form appears transparent, retains its scattering ability, and has the added bonus of greater relative surface area allowing superior UV-absorbing capacity [38]. For these reasons, nanoparticles are more commonly used today in sunscreen formulations.

**Figure 2:**
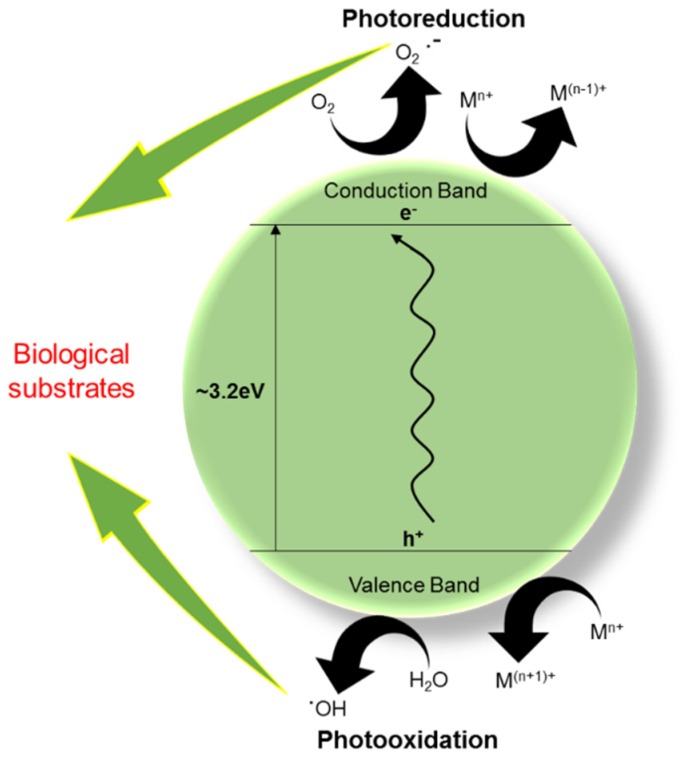
The semiconducting and photocatalytic properties of TiO_2_ NPs.

The UV protection provided by TiO_2_ in sunscreen stems from its function as a semiconducting material. TiO_2_ is an intrinsic N-type semiconductor due to oxygen vacancies in its lattice [39]. It is generally characterized by the band gap energy of ∼3.2 eV [40]. In its anatase form, the band gap corresponds to a wavelength of 387 nm and in its rutile form, it is 405 nm. Light at or below these wavelengths can excite electrons from the valence band to the conduction band (Figure 2) [41]. In sunscreen, TiO_2_ NPs absorb the UV-radiation from the sun by promoting electrons from its valence band to its conduction band [42]. This process results in photogenerated holes in its valence band. The photogenerated holes and excited electrons (Equation (1)) can either recombine or migrate to the particle surface and participate in different redox processes that lead to the formation of reactive oxygen species (ROS) (Equations (2)–(5)) [42]. Being a powerful oxidant, the valence band holes primarily target moisture present on the surface (Equation (2)), which produces hydroxyl radicals. The conduction band electrons are good reductants and for them, oxygen present at the surface acts as a primary electron acceptor producing superoxide and eventually hydrogen peroxide (Equation (3)) [43]. The conduction band electrons could also be rapidly trapped at Ti(IV) sites and then react with oxygen, yielding superoxide and hydrogen peroxide (Equations (4) and (5)) [43,44].

(1) $${\mathrm{TiO}}_{2}\;+\;h\upsilon \;⟶\;(e_{CB}^{-}\;+\;h_{VB}^{+})\;{\mathrm{TiO}}_{2}$$

(2) $$h_{VB}^{+}\;+\;H_{2}O\;⟶\;H^{+}\;+•\;\mathrm{OH}$$

(3) $$e_{CB}^{-}\;+\;O_{2}\;⟶\;{O_{2}}^{•-}\;⟶⟶⟶\;H_{2}O_{2}$$

(4) $$e_{CB}^{-}\;+\;\mathrm{Ti}(\mathrm{IV})\;⟶\;\mathrm{Ti}(\mathrm{III})$$

(5) $$\mathrm{Ti}(\mathrm{III})\;+\;O_{2}\;⟶\;\mathrm{Ti}(\mathrm{IV})\;+\;{O_{2}}^{•-}\;⟶⟶⟶\;H_{2}O_{2}$$

If left uncontrolled, the ROS formed can target different substrates and be responsible for biological impairments. They can cause oxidative stress within cells including DNA damage, modification of proteins and sensitive thiols, and trigger redox activity of copper and iron ions [42,45,46,47,48]. Within sunscreen, radical scavengers and antioxidants are included or are coated on the surface of TiO_2_ to suppress TiO_2_-induced ROS generation and prevent harm to human skin [49]. Due to the strong redox reactivity of TiO_2_, there has been great interest in its use as a photocatalyst for energy and biomedical applications, namely as anticancer [50] and antibacterial agents [51] (See Section 4).

## 3. The Biological Use and Solubility of TiO_2_ and Its NPs

TiO_2_ is structurally extremely stable especially in the anatase, brookite, and rutile crystalline forms [52], and exhibits very poor aqueous solubility [53]. These properties are key to the application of TiO_2_ in sunscreen and other human cosmetics. Schmidt and Vogelsberger performed an extensive study to examine the aqueous solubility of TiO_2_ in crystalline and amorphous hydrous forms under conditions that are physiologically relevant [54]. They found that crystalline forms are significantly less soluble under acidic pH and result in Ti(IV) concentrations of about 1 nanomolar in the pH 4 to 10 range. This finding suggests that TiO_2_ NPs should be virtually insoluble in sunscreen particularly because of the water-resistant formulations of the sunscreen and, thus, not cause toxicity on account of dissolution-related phenomenon. Lack of solubility would retain the very high stability of these materials. It has long been thought that TiO_2_ is biologically inert. 

Several lines of evidence suggest that in a biological context, TiO_2_ is active. In diatoms, an amorphous coating of it can be found on the SiO_2_ crystalline lattice of the frustules. The diatoms are believed to take advantage of the photocatalytically activated antibacterial properties of TiO_2_ to ward off predators [55]. TiO_2_NPs have demonstrated many benefits to plant growth, some of which appear to be species specific. They can increase seed germination rates and seedling growth, enhance root lengths, improve plant growth, and increase crop growth and yield [56]. They can also increase plant tolerance to abiotic and biotic stresses, including cold stress, heat stress, drought, and cadmium toxicity [56]. Additional benefits have been enhanced photosynthesis, increased chlorophyll content, and exploiting the photoactive antibacterial properties of TiO_2_ to control bacterial and fungal pathogens in crop production [56]. A micro-X-ray absorption near edge structure (micro-XANES) study with a cucumber plant *Cucumis sativus (C. sativus*) showed a varied biodistribution of TiO_2_ throughout the plant following treatment with a mixture of 19% rutile and 81% anatase [7]. In the xylem, the % of both rutile and anatase remained fairly consistent with the bulk material but within the phloem, TiO_2_ was exclusively rutile. It was reasoned that the size of anatase restricted it from uptake beyond the roots. The TiO_2_ improved the plant’s growth possibly because of nitrogen activation [7] as a result of coordination to the metal.

Although there is no known function for Ti in bacteria, certain bacteria have the capacity to interact with the metal in TiO_2_ form. The chemical proximity of Ti(IV) with iron(III) (Fe(III)) [33] could account for bacterial interaction with Ti as bacteria heavily depend on Fe for survival and have evolved numerous acquisition pathways for mobilizing and capturing the metal [57]. *Rhodococcus ruber (R. ruber)* GIN1, a Gram-positive species, strongly adheres to TiO_2_ under a wide pH and temperature range [58]. A 52 kDa TiO_2_-binding protein was isolated from the bacteria and identified to be a cell surface form of dihydrolipoamide dehydrogenase (rhDLDH) [59]. It adheres more effectively to the rutile than the anatase form [59]. A human homolog exists, which at pH 8.0, binds to TiO_2_ and other metal oxides (ZnO, MgO, MnO, Al_2_O_3_, Fe_2_O_3_) [60]. The affinity is highest for TiO_2_ and Fe_2_O_3_ [60]. The binding interaction between RhDLDH and hDLDH with metal oxides appears to be nonelectrostatic. A computational study was performed to identify the binding site for TiO_2_ and this led to the determination of the involvement of a putative CHED motif (Cys, His, Glu, Asp) in both enzymes. CHED motifs are known to coordinately bind metal ions but how this motif coordinates to the Ti in TiO_2_ is not yet known [60]. Very recently, hDLDH was coated onto the TiO_2_ surface layer of a Ti-containing implant to enhance the osseointegration property of the implant [61]. The use of a coating of a human protein is expected to enhance the biocompatibility of the implant surface. 

**Figure 3:**
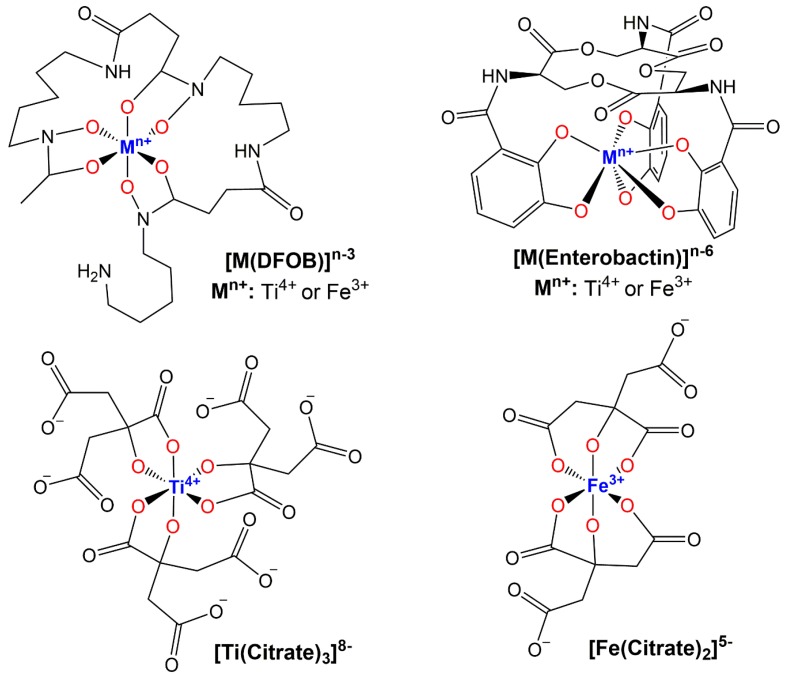
Coordination similarities of siderophore binding of Ti(IV) and Fe(III) at pH 7.4. The hexadentate siderophores, Desferrioxamine B (DFOB) and Enterobactin, form 1:1 metal:ligand complexes. Citrate coordinates Ti(IV) and Fe(III) in slightly different ways. Citrate serves as a tridentate ligand when coordinated to Fe(III) and as a bidentate ligand when coordinated to Ti(IV).

Other bacteria such as *Escherichia coli* (*E. coli)* and *Pseudomonas aeruginosa (P. aeruginosa)* have also been observed to interact with TiO_2_ NPs with cell surface siderophores [62,63,64]. Siderophores are low molecular weight molecules that organisms (bacteria, yeast, algae, plants) synthesize and export to mobilize and/or sequester Fe(III) through different molecular pathways [57]. They consist of the functional groups catechols, hydroxamic acid, and α-hydroxy-carboxylic acids. The siderophore pyoverdine can enable *P. aeruginosa* to simultaneously bind to TiO_2_ and iron oxide through direct coordination of the metal by its catechol moiety. It is speculated that the oxides provide a template for biofilm formation [62]. Siderophores can tightly bind Ti(IV) in ion form, producing coordination complexes comparable to their Fe(III) counterparts at physiologically relevant pH values (Figure 3). Enterobactin contains three catechol moieties that coordinate Ti(IV) in hexadentate fashion forming a 1:1 metal:ligand complex, the crystal structure of which has been reported [65]. Desferrioxamine B (DFOB), a trishydroxamic acid siderophore, also coordinates Ti(IV) in a hexadentate modality. Valentine et al. reported a density functional theory (DFT)-optimized structure for the 1:1 metal:ligand complex and explored its aqueous speciation over the pH 2 to 10 range [66]. Citric acid is considered a siderophore with α-hydroxy-carboxylic acid moieties [67]. There are many examples and structures of Ti(IV) citrate complexes, in which the ligand binds in bidentate fashion and can coordinatively saturate the metal [31,33,68,69,70,71,72,73]. It is important to note that citrate binds Fe(III) in a slightly different fashion by coordinating in tridentate mode using one of the carboxylic acid groups in the β position to provide the extra coordination site [67,74]. Valentine made the very important discovery that siderophores can dissolve TiO_2_ by a coordination-induced mechanism [75]. The details of this study are pending but the work suggests that bacteria could mobilize Ti(IV) in ways comparable to its acquisition of Fe(III) and therefore transform the metal into a bioavailable species that may serve a function.

Biomolecular solubilization of TiO_2_ suggests that the metal oxide might be far more soluble than previously considered in human products. Several skin care products, including sunscreen, contain hydroxyacids like salicylic acid and citric acid that can chelate Ti(IV) [31,33,68,69,70,71,72,73,76] and potentially lead to its dissolution. One study examined the solubility of TiO_2_ in rutile and anatase form in an oil in water (o/w) weakly acidic emulsion that mimics cosmetic formulations. It was determined that after 1 to 2 days of 1 g TiO_2_ suspended in 0.03% (w/w) citric acid mixed in the o/w emulsion, ~600 μM Ti(IV) was found present in solution. This is several orders of magnitude higher than the innate solubility of TiO_2_. In human blood and synovial fluid (pH 7.4), citric acid is present as citrate in the concentration range of 100–200 μM. It is believed to contribute to the solubilization of some of the metal leached from Ti-containing implants by forming, at least transiently, the coordination complex Ti(IV) tricitrate. The complex is able to deliver Ti(IV) to the two metal binding sites of serum transferrin [31]. One citrate molecule can remain bound to the Ti(IV) at each site, enhancing the stabilization of the metal ion [31]. The interaction of citrate and sTf in regulating the blood speciation of Ti(IV) may account for the Ti blood levels of ≤0.25 μM in people with these implants, levels that are not expected to be toxic [19]. Citrate and sTf are hypothesized to engage in a synergistic molecular mechanism to decrease the cytotoxic properties of some anticancer Ti(IV) complexes by inducing their dissociation and scavenging the metal [31,77]. It is unknown whether Ti(IV) solubilization from skincare products, if it does occur in reality, could lead to elevated levels of the metal in the human body. 

## 4. Applications of TiO_2_ NPs Provide Further Insight into Its Functionality

TiO_2_ NPs are one of the most manufactured nanomaterials worldwide with an estimated annual median of 3000 tons produced [78]. Of this total, 70%–80% of it is used in the cosmetic industries, which includes sunscreens [78]. There is a significant level of daily dietary intake of TiO_2_ NPs in human beings because it is used as a whitening agent of certain foods [79]. Extensive production and growing applications are responsible for its exposure to the environment. In the United States, the daily consumption of TiO_2_ has been estimated in the range of 0.2–2 mg/kg of body weight and per day [80]. During bathing activities, TiO_2_ NPs enter the water bodies. A research group determined that the concentration of titanium ranges from 181 to 1233 µg/L in raw sewage obtained from 10 full-scale municipal centralized wastewater treatment plant municipalities in Arizona [81]. The treated water from these water plants flows into rivers and lakes where nanoparticles may cause an ecological risk. It has been found that the released TiO_2_ NPs in the water bodies stay at the air–water interface for a short time and float on the water surface or hetero-aggregate with natural suspended particulate matter and sediment [82].

Despite its emerging status as a contaminant in water bodies, TiO_2_ NPs is extensively studied as a material for several photocatalytic applications. Efforts are being directed at maximizing capturing the energy from sunlight. Sunlight’s emission spectrum consists of only 4% UV light, whereas visible light constitutes a significantly larger percentage, approximately 40%. As documented in a recent review by Tan et al., the photocatalytic properties of TiO_2_ NPs can be fine-tuned by structurally modifying it to decrease its bandgap in order to more effectively utilize visible light [83]. This can be achieved by introducing additional intrinsic defects, doping with a range of non-metal elements, shielding the particle through a suitable coating, by functionalizing the NPs, and testing different particle sizes [34,41,83,84,85]. 

A specific photocatalytic application of TiO_2_ NPs being explored is sterilization because of its effectiveness in treating a wide variety of pollutants (e.g., pharmaceuticals, pesticides, antibiotics, endocrine-disrupting compounds), food, and bacteria. Carbamazepine is an antiepileptic pharmaceutical compound that is frequently found in water bodies and is believed to be a danger to aquatic life including bacteria, algae, invertebrates, and fishes, etc. [86]. It cannot be efficiently removed (<10%) by conventional wastewater treatment plants. Several studies have shown that it can be photodegraded using TiO_2_-suspended NP photocatalysts [87,88,89]. Salicylic acid, another pollutant, can be degraded when subjected to UV irradiation in a photocatalytic reactor that uses TiO_2_ NPs as a semiconductor [90]. 

The photocatalytic bactericidal effect of TiO_2_ NPs has been an emerging field of investigation since the early 1990s [91,92,93]. In a study, researchers designed a photobioreactor to sterilize the selected foodborne pathogenic bacteria, *Salmonella choleraesuis (S. choleraesuis)*, *Vibrio parahaemolyticus (V. parahaemolyticus)*, and *Listeria monocytogenes* (*L. monocytogenes)* using various TiO_2_ NP concentrations and ultraviolet (UV) illumination time [94]. The survival of all bacteria was decreased to ~20%–60% in the presence of UV-radiated TiO_2_ NP (1.00 mg/mL) within 30 min. of illumination. Currently, research is focused on photoinactivation of various bacterial strains using doped TiO_2_ NP photocatalysts, which include several different doping systems, for instance, nitrogen, silver, manganese, zinc oxide, sulfur, nickel, copper, and silicon [95,96,97,98,99,100,101,102]. The bactericidal mechanism is well characterized. The damage starts via bacterial cell membrane disruption caused by ROS, which results in the subsequent leakage of internal components from the damaged sites [103,104,105]. 

Investigations of TiO_2_ NP use for their photocatalyzed anticancer properties has been another area of major interest. Zhang et al. have studied the photocatalytic killing effect of TiO_2_ NPs on colon carcinoma cells and concluded that at concentrations lower than 200 µg/mL, they are effective protection against UVA irradiation alone, but above this concentration, there is a significant cytotoxic effect on these cells [106]. The mechanistic details underlying this cytotoxic behavior has been studied. Wamer et al. observed that nucleic acids are the main target for photooxidative damage catalyzed by TiO_2_ NPs. They observed the hydroxylation of guanine bases following calf thymus DNA reaction with TiO_2_ NP while irradiated with UVA [107]. Jaeger et al. examined the putative pathway for TiO_2_ NP-induced mitochondrial DNA damage in human HaCaT keratinocytes [108]. They found that ROS generation resulted in the mitochondrial common deletion of DNA base pairs in HaCaT cells. Lagopati et al. saw a significant induction of apoptosis in MDA-MB-468 cells when they irradiated the breast cancer epithelial cells using UV-A light (wavelength 350 nm) for 20 min in the presence of nanostructured TiO_2_ sol-containing anatase NPs [109]. 

The utility of TiO_2_ NPs as photosensitizers in targeted anticancer photodynamic therapy (PDT) is being explored. Zhang et al. compared the photosensitizer capacity for TiO_2_ NPs with that of ZnO NPs and observed no differences in their anticancer potencies as both could generate ROS and lead to caspase-dependent apoptosis within the tumor cells [110]. Current research is more focused on the development of modified TiO_2_ NPs to enhance their photocatalytic activity. Yang et al. synthesized Ce-doped TiO_2_ nanocrystals by a modified sol-gel method for the treatment of deep-seated tumor [111]. These nanocrystals could serve as photosensitizers in PDT when activated by low-dose X-ray as they can generate intracellular ROS and lead to the apoptosis/necrosis of A549 cancer cells. The use of TiO_2_ NPs as photosensitizers for photodynamic antibacterial therapy is also being investigated [112,113].

## 5. Elucidating the Impact that the Bioactivity of TiO_2_ NPs from Sunscreen Use Could Have on the Aquatic Environment and Human Health

Industrial applications of photoexcited TiO_2_ NPs demonstrate their potent redox reactivity and hint at the effect that they may have on the biological activity in prokaryotic and eukaryotic cells. TiO_2_ NPs released into the environment could lead to the toxicity of aquatic organisms. Mueller and Nowack determined a predicted no effect concentration (PNEC) of <1 μg/L for TiO_2_ exposure to aquatic organisms such as algae and daphnia [114]. Below this value, the TiO_2_ content in water bodies is not expected to cause any toxicity. In 2010, the Environmental Protection Agency (EPA) issued a case study on nanoscale TiO_2_ and its use in topical sunscreen [38]. It evaluated the stability of the material and its safety to the environment and people without making any definitive statements in support or against use of the material. The study revealed that at very high concentrations (in the mg/mL range), TiO_2_ NPs is toxic to several algae, invertebrate organisms, and fish, a predictable result considering that the content far exceeds the PNEC value [38]. Ates et al. investigated the bioaccumulation and tissue distribution of TiO_2_ NPs in goldfish (*Carassius auratus (C. auratus)*) [115]. In the study they found that a short period of exposure to 10 and 100 mg/L concentrations of TiO_2_ NPs was not lethal, however physiological and behavioral changes were noticed at exposure to higher concentrations. The accumulation of TiO_2_ NPs in the intestine was increased when the concentration of NPs was increased from 10 to 100 mg/L; conversely, the weight-wise growth of goldfish was decreased at higher concentrations. Mansfield et al. demonstrated the photo-induced toxicity of anatase TiO_2_ NPs under natural sunlight to small planktonic crustacean *Daphnia magna (D. magna)* [116]. They determined the LC_50_ for NPs after 8 h of sunlight exposure. Under full intensity ambient natural sunlight, the LC_50_ was 139 ppb, under 50% natural sunlight, the LC_50_ was 778 ppb and >500 ppm under 10% natural sunlight. Kachenton et al. investigated the toxicological effects of TiO_2_ NPs to the brine shrimp (*Artemia salina (A. salina)*), by determining 24 h LC_50_, which was 1693.43 mg/L. Jovanovic et al. have conducted a series of studies probing the different types of detrimental effects that TiO_2_ NPs can have on aquatic organisms. In one such study, they exposed Caribbean mountainous star coral (*Montastraea faveolata (M. faveolata)*) for 17 days in 0.1 mg/L and 10 mg/L TiO_2_ NP suspensions. The coral exhibited symptoms of acute stress including expulsion of zooxanthella and a temporary increase in the expression of the heat-shock protein 70. In addition, bioaccumulation of the NPs was observed in the microflora of the coral [117]. In another study, Jovanovic examined the immunotoxicity of fish (*Fathead minnows*; *Pimephales promelas (P. promelas)*) induced by TiO_2_ NPs [118]. Due to their antibacterial properties, the NPs were expected to serve as protective agents against predatory bacteria. However, they caused a reduction in the antibacterial activity of fish neutrophils (which function to eliminate bacteria by phagocytosis), histopathological effects, and an increase in mortality when challenged with two bacterial strains [118]. This suggests that fish with elevated levels of TiO_2_ NPs would be liable to bacterial infection and increased mortality during disease outbreaks.

Many of the studies that report on the dangers of TiO_2_ NPs focus on effects at high concentrations that may not reflect physical reality. The EPA reports that a number of environmental factors can contribute to the perceived effects of the NPs such as the UV index, the pH and chemical composition of a body of water, ambient temperatures, and ecological factors such as the storage and potential seepage of wastewater containing the NPs [38]. In addition, the size, crystallinity (whether anatase, rutile, or other forms), and surface coating of the NPs have a major influence [38]. Considering all of these factors is outside the scope of this work but they certainly impact the solid and solution state speciation [16,17,18] of the metal and its reactivity. Reports that focus on direct measurements of TiO_2_ NPs in bodies of water provide a more realistic perspective on the safety of TiO_2_ NPs. There is on average 46 mg of TiO_2_ NP content present in per gram of sunscreen with an adult application of about 36 g, from which 25% of the total applied could wash off from the skin in the water during beach activities [119]. Sánchez et al. estimated the summer daily release of TiO_2_ NP of approximately 4 kg at Palmira beach (Peguera, Majorca Island) and estimated an associated increase of net hydrogen peroxide production rate of H_2_O_2_ of 270 nM/day due to the redox activity of the material [119]. Venkatesan et al. used single-particle inductively coupled plasma mass spectrometry to measure Ti-containing particles in heavily frequented bathing areas in Arizona—the Salt River and five swimming pools [120]. Between 64 to 148 ng/L of TiO_2_ NP were found in the pools whereas 260–659 ng/L were found in the Salt River, this number bordering very close to the PNEC limit. The concentration range for the Salt River is expected to be underestimated value because it is possible that larger-sized particles may have been filtered out in the sample preparation process and smaller-sized particles are undetectable by the instrument [120]. These particles are believed to originate from sunscreen products as TEM images compare favorably with Ti-containing NPs from commercially available sunscreen. Interestingly, the Ti content in the swimming pools was dominated (98.7%–99.8%) by dissolved Ti species [120]. Holbrook et al. also made a similar observation of dissolved Ti species in a swimming pool [121]. The source of this dissolved Ti and its speciation has not been characterized. 

Whether TiO_2_ NPs can dissolve in open water has not been established. There are a number of organisms that possess biomolecules with chelating moieties that have the capacity to bind Ti(IV) due to its hard Lewis acidic nature [33,122], such as siderophore-producing marine organisms [67], the dihydroxyphenylalanine (DOPA)-containing adhesive proteins of mussels [123,124,125], and the tunichromes of ascidians [126,127]. That said, whether chelation onto the metal in TiO_2_ NPs can induce solubility needs to be examined. There are marine organisms like the brown algae *Fucus spiralis (F. spiralis)* (308 ppm) [128] and the ascidian *Eudistoma ritteri* (*E. ritteri)* (1512 ppm) [129] that can bioaccumulate Ti(IV) at several orders of magnitude greater than their local environment. This elevated concentration is presumably the product of biomolecular chelation although the reason for this binding has not been established. It is possible that the Ti(IV) is functionally useful to these living things. The profile for dissolved titanium in the open ocean suggests that the metal is biologically used. Its surface concentrations are quite low where there is an abundance of living organisms but are significantly higher at greater depths where life is less prevalent [130,131]. Were TiO_2_ NPs to become solubilized in open waters, then perhaps the soluble Ti(IV) may not be too much of a concern if there are marine organisms that can scavenge and potentially utilize the metal unless the solubilized levels become too extreme or the speciation toxic. 

**Figure 4:**
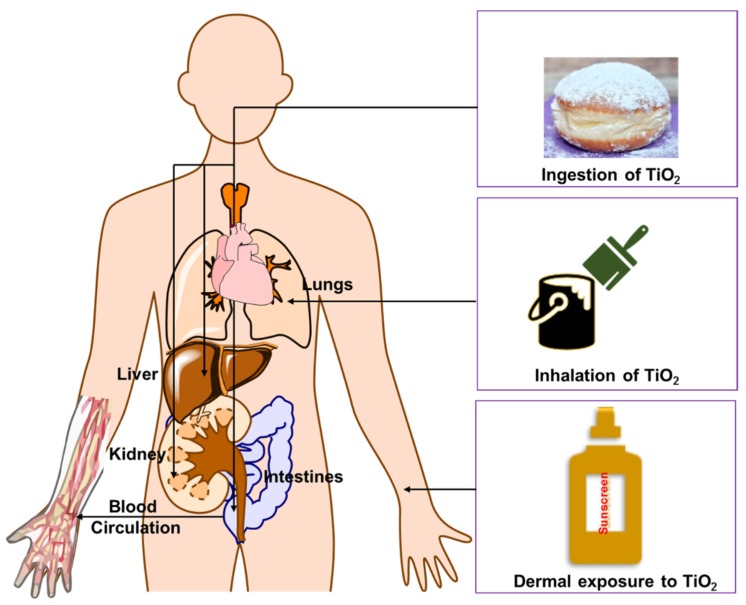
Different pathways by which TiO_2_ nanoparticles can enter and distribute in the human body.

A major issue for debate is the long-term effect of TiO_2_ NPs on human skin and human health, in general, from sunscreen use. To address this matter, it is important to distinguish between potential effects from the NP form of these materials and any solubilized Ti(IV). Before doing so, let us consider routes of entry into the body. It is generally accepted that TiO_2_ NPs and Ti(IV) ions can enter the human body primarily through inhalation (respiratory tract) and ingestion (gastrointestinal tract), the latter of which can lead to its circulation in blood [132] (Figure 4). It is much less clear how and if it actually penetrates the skin. Mammalian skin is structured in several layers: The stratum corneum (SC), epidermis, dermis, and the subcutaneous layer. SC is the rate limiting barrier against absorption/percutaneous penetration of topically applied substances [133]. The epidermis, the outermost layer of the skin, works as a barrier for the dermis, which contains connective tissue, sweat glands, hair follicles, and nerve endings. Some evidence suggests that TiO_2_ NP may penetrate into or through human skin and can reach to the epidermis or dermis [132]. Most studies indicate that TiO_2_ NP penetration is localized within the SC and hair follicles and much less penetration occurs at the epidermis or dermis. Sadrieh et al. showed that repeated application of 5% TiO_2_ uncoated or coated particles of a 20–500 nm size range can penetrate the skin of mini pigs, leading to detectable levels of the particles in the dermis. It was unclear whether the presence of NPs in the dermal part of the skin resulted from viable skin penetration or from their presence in the hair follicles. The study of long-time (60 days) exposures of 4 and 60 nm TiO_2_ NPs on hairless mice showed deeper penetration of TiO_2_ [134]. The NPs were allocated in various tissues such as the lungs, spleen, and brain, indicative of potential crossing of the blood–brain barrier. Of the organs examined, the skin and liver exhibited the most severe pathological lesions, which are believed to be due to the oxidative stress caused by the NPs [134]. This work suggests that after repeated (long-term) application of sunscreen, TiO_2_ NPs contained within may be able to translocate through human skin. 

The interaction of the nanomaterials with macromolecules, for example, proteins is an interest for several points of view. The realization of this synergistic effect on nanotechnology and its subbranches (nanobiotechnology, protein nanotechnology, nanomaterial science, etc.) especially in improving the performance of proteins and enzymes for various applications, is evident from the numerous studies that have emerged in the last few years. A large emphasis is given to improving the performance of proteins when they are formulated in combination with other nanomaterials and, therefore, the interaction between proteins and nanomaterials are widely examined. However, despite the significant advancement in this area, comparatively less focus has been given to cellular uptake studies of nanomaterials facilitated by proteins in biological systems. Therefore, exact uptake pathways, mechanisms, and the final effect of nanomaterials inside the cell are poorly recognized. Nevertheless, some recent reports in this direction provide elucidate important insight, which is helpful not only for the smart utilization of the nanomaterial but also in testing biological responses towards nanoparticles and dose-dependent toxicity. The increasing use of TiO_2_ NPs and, more recently, the discovery of the degradation of titanium implants resulting in the formation of said particles, has raised questions about its fate and effect in mammalian organisms. Several studies involving the injection and subsequent tracking of TiO_2_ NPs in rats have discovered the deposition of these particles into the spleen, liver, and lung tissues. Although these studies linked the transportation of TiO_2_ NPs to macrophages, the exact mechanism of uptake in these cells was not probed [135,136]. An important aspect to consider in the uptake of TiO_2_ NPs is the formation of the protein corona (PC), a layer of proteins that rapidly covers nanoparticles when present in a protein-rich environment such as the human serum. The PC mediates the interactions between the NPs and cells, and so its composition plays an important role in the translocation of TiO_2_ NPs [137]. 

Several blood and serum proteins have been studied so far to understand the effect of individual protein/s through cellular uptake routes such as phagocytosis, micropinocytosis, or endocytosis [137]. Serum/plasma proteins constitute a complex proteome system where different proteins will interact with nanoparticles at different physiological conditions (pH and ionic strength) and, therefore, injected nanoparticles might undergo modifications, which are not completely identified [137]. In a study by Tedja et al., TiO_2_ NP uptake into the human lung cell lines A549 and H1299 was investigated with an emphasis towards PC function and composition [138]. By treating TiO_2_ NP with fetal bovine serum (FBS), a protein-rich serum, and comparing it to the uptake of non-treated NPS, some insight was gained into the role of the PC. The size of the TiO_2_ NPs suspended in FBS was smaller in comparison to those in PBS buffer alone. The reduction in the particles size is attributed to the coating of complex proteins-mix on the surface of the particles, which reduce aggregation by forming a steric layer. Similar results were observed by Allouni et al. by using anatase TiO_2_ NPs adsorbed on three blood proteins; human serum albumin, γ-globulins, and fibrinogen for uptake studies using L929 mouse fibroblasts cells [139]. Although there was a larger initial uptake of the non-FBS-treated TiO_2_ NPs, after a 24 h period, the FBS-treated particles showed a larger uptake into the cells, which was attributed to a second phase of particle uptake observed in the data [138]. Additionally, a difference in uptake was observed between the two cell lines, with the H1299 having a higher uptake than the A549 cells, highlighting the difference in biochemical composition of the cell membranes and consequently a difference in cellular uptake in cells from the same tissue of origin. Apart from looking at the TiO_2_ NP uptake of these cell lines, its pathway was also probed. By subjecting the cells to cellular uptake inhibitory treatments, namely low temperature, adenosine triphosphate depletion, caveolae disruption by cholesterol sequestration, and hypertonic treatment, they were able to ascertain endocytosis as the main uptake mechanism in both cell lines. Furthermore, the data obtained suggested endocytosis in the A549 occurred via a clathrin-mediated pathway, while the H1299 uptake mechanism remained elusive. Another important aspect covered in this study was the potential role of the serum protein vitronectin, a serum glycoprotein associated with cellular adhesion to surfaces and the uptake of crocidolite asbestos in rabbit pleural mesothelial cells and A549 cells via the α_v_β_5_ integrin receptors [140,141,142]. By using an antibody to remove vitronectin and measuring the uptake of TiO_2_ NP into cells, the authors were able to pinpoint vitronectin as an important factor for TiO_2_ NP absorption in A549 cells [138]. Although this effect was not observed in H1299 cells, this last result highlights the important role that PC composition plays in the interaction between TiO_2_ NPs interaction with cells and their subsequent uptake. 

Toll-like receptors (TLR3, TLR4, and TLR7) have also been studied in the uptake of TiO_2_ NPs. These receptors are transmembrane proteins, which play an important role in cellular defense, ligand binding, and signaling pathways [143]. TLR4 and TLR7 were found to be able to transport the NPs [143]. The involvement of TLR4 and other various receptors and proteins in uptake pathways was also investigated in the sea urchin *Paracentrotus lividus (P. lividus)* immune cell [144]. High expression of the TLR gene and in the levels of related proteins in immune cells was observed when TiO_2_ NPs encounter the immune cells. The size of the NPs is important for such uptake studies as the NP size generally falls within the range of bacteria and fungi, which is perhaps related to identifying the foreign material [144,145]. 

Some studies have moved toward PC characterization, and have identified serum and plasma proteins such as immunoglobulin, human serum transferrin (hTf), and human serum albumin (HSA) in the TiO_2_ PC [146,147,148]. Due to its metal-binding ability and abundance in human serum, HSA and its interaction with TiO_2_ NPS has been extensively studied. It has been shown that in different media, the presence of the HSA model protein, bovine serum albumin (BSA), FBS, or a mixture of serum proteins (BSA, γ-globulin, and apo-hTf) improved the dispersion of TiO_2_ NPS. This study, which used high-throughput dynamic light scattering (HT-DLS) to determine nanoparticle size distribution and state of agglomeration-dispersion, was able to observe the effect of the PC on the hydrophobic and electrostatic interactions, which govern TiO_2_ aggregation or dispersion. In almost every case, BSA aided in TiO_2_ dispersion but most noteworthy is the synergistic effect observed in the FBS and the mixture of serum proteins, which had a higher stabilizing effect on all culture media, highlighting the important role of the diversity of the PC in stabilization of TiO_2_ NPs [149]. Due to the nature of the interactions governing PC formation, many factors have to be taken into account. Environmental factors such as pH and salt content have been shown to have an effect in the binding of HSA and possibly other serum proteins, mainly due the protonation/deprotonation of the TiO_2_ NPs surface and the change in protein charge induced by the pH [149,150]. Additionally, the morphology of the particles has been shown to be an important factor in protein–NPs interaction. Zaquot et al. studied the binding of serum proteins to the anatase, rutile, and polymorph forms of TiO_2_ and found that the polymorph form had a greater adsorption of serum proteins, although the exact mechanism of these interactions could not be determined [151]. The complex nature of the PC and its interaction with TiO_2_ NPs makes it difficult to study in vivo, although they are certain to be involved in its transport through the blood stream and translocation to systemic organs [135,136]. Despite their clear limitations, in vitro studies have proven to be useful in observing these interactions and help us work towards an understanding of TiO_2_ NP–protein interactions and transport into the cells and through the human body.

Whether or not TiO_2_ NPs from sunscreen use are able to penetrate the skin, it is important to consider the different effects that they might have on human cells. As already established, by carefully regulating their redox activity, TiO_2_ NPs can be designed as potentially excellent anticancer agents. However, the absence of control over this activity could lead to many issues to cells and tissue. The excessive generation of ROS and reactive nitrogen species (RNS) could lead to inflammation, fibrosis, and pulmonary damage [152]. At the cellular level, oxidative stress can occur, resulting in chemical and structural modifications of macromolecules and interference with signal transduction pathways and changes to transcription factors. In extreme cases, oxidative DNA damage occurs that results in cytotoxicity or in mutations that can cause cancer. Jin et al. observed that following treatment of mice fibroblast cells (L929) with colloidal TiO_2_ NPs (3–600 μg/mL), the cells became round, shrank, and lost their ability to adhere to one another and to proliferate. They exhibited severe DNA damage from oxidative stress and were either in the late stages of apoptosis or necrotic. The TiO_2_ NPs appeared to alter the release and structural composition of lysosomes, which in turn, led to increased lysosomal permeability and to damage and destruction of organelles, to changes in mitochondria, and to triggering of apoptosis [153]. Concerns regarding the cytotoxicity of TiO_2_ NPs may be more directed at human skin and layers beneath the surface to the extent at which UV light can penetrate although the NPs can certainly produce oxidative stress at high concentrations, without UV activation. As for the cancer-causing ability of TiO_2_ NPs, the International Agency for Research on Cancer, which is part of the World Health Organization, classified it as carcinogenic to animals [48]. The evidence from epidemiological studies is extremely poor for classifying the NPs as carcinogenic to humans. However, it is labeled as potentially carcinogenic to humans and this potential appears to be targeted to lungs [48]. TiO_2_ is found at its highest levels in the lungs of the human body (3.7 μg/g of body) due to the ubiquitous nature of TiO_2_ particles in the air [29,154]. A single multi-country study of TiO_2_ NP production workers found that the workers had higher TiO_2_ levels and a slightly higher risk for lung cancer than the general population and exhibited a clear dose-response to inhalation exposure [48]. For this reason, the use of spray-on sunscreen that contains TiO_2_ NPs is not recommended because it can lead to increased inhalation of the particles [38,48]. Throughout the world, the safety classification of TiO_2_ NPs is greatly debated. Very recently (October 2019), France was the first nation to publish a law suspending the use of food additive TiO_2_ (E171) due to its numerous health risks. Several non-governmental organizations, including the European Environmental Bureau (EEB), ClientEarth, and the Center for International Environmental Law (Ciel) are pushing for similar laws within the European Union to classify TiO_2_ NPs in all of its forms as a category 2 carcinogen. In contrast, the U.S. Food and Drug Administration (FDA) recently evaluated the physicochemical properties, reactivity, and skin contact mobility of TiO_2_ NPs and deemed their use in over-the-counter sunscreens as generally recognized as safe and effective (GRASE) [155]. 

**Figure 5:**
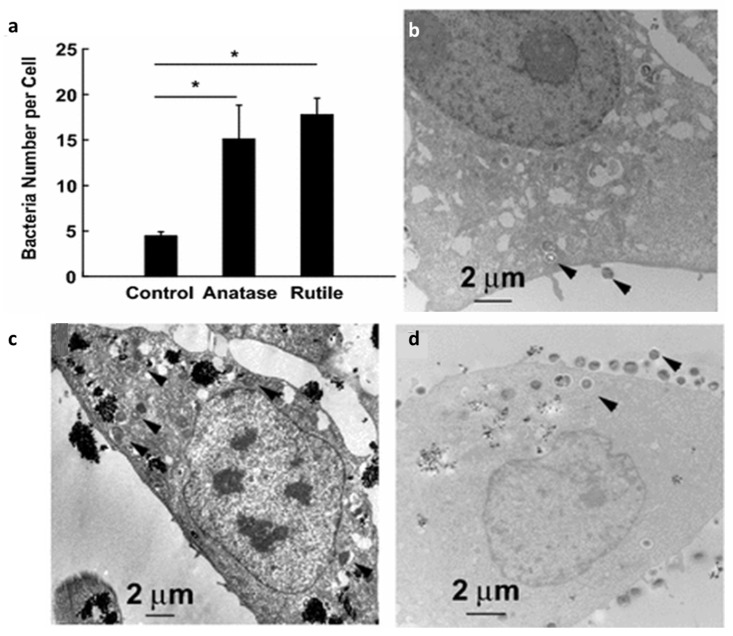
The effect of TiO_2_ NPs on infectious bacteria *S. aureus* in HeLa cells. The number of bacteria *S. aureus* in control vs. anatase and rutile exposed HeLa cells (**a**). TEM cross-sections of HeLa control cells (**b**), cells exposed to 0.1 mg/mL anatase (**c**), and 0.1 mg/mL rutile TiO2 (**d**) followed by exposure to *S. aureus* bacteria for 90 min. Arrows point towards bacteria. * Means *p* < 0.05. This figure was modified from *Journal of Nanobiotechnology*, 14:34, Copyright 2016, Springer Nature. This work was published under a CC BY 4.0 license (http://creativecommons.org/licenses/by/4.0/) [156].

There are additional safety factors that should be evaluated. In the absence of UV activation, TiO_2_ NPs can still have a debilitating effect on human cells. UV activation enables the NPs to exhibit prophylactic activity against bacteria as previously described but surprisingly, sans the UV energy, they double the risk of human cells for bacterial infection similar to what has been reported for fish [118]. Mironava et al. examined the non-UV effect of TiO_2_ NP treatment of human cervix adenocarcinoma (HeLa) cells at levels that do not induce ROS and in the presence of *Staphylococcus aureus (S. aureus)* [156]. This gram-positive bacteria can be found on human skin. While there was no observed change in cell size, the HeLa cells were more porous when treated with anatase and rutile than normal and showed loss of membrane integrity. Both anatase and rutile caused a similarly elevated level of bacteria in the HeLa cells compared to control (Figure 5) but, unexpectedly, not in macrophages. This suggests that they induce a compromised immune response, comparable to findings with fish [118]. The increased porosity of human cells by TiO_2_ NPs leads to the release of lactate dehydrogenase, which Mironava et al. believe produces a favorable extracellular environment that bacteria may be attracted to [156]. It does not appear as though the higher attraction for these cells is because the bacteria are drawn to the Ti itself, but this factor needs to be examined in light of evidence that bacterial biomolecules can interact with Ti(IV) (See Section 3). In a related work, Wang et al. found that TiO_2_ NP dietary exposure induced an abnormal state of macrophages characterized by excessive inflammation and suppressed innate immune function [157]. The macrophages exhibited decreased chemotactic, phagocytic, and antibacterial activity, which make people more susceptible to infections. Piedimonte et al. have demonstrated the enhanced susceptibility to respiratory syncytial virus infections in human bronchial epithelial cells exposed to TiO_2_ NPs [158].

**Figure 6:**
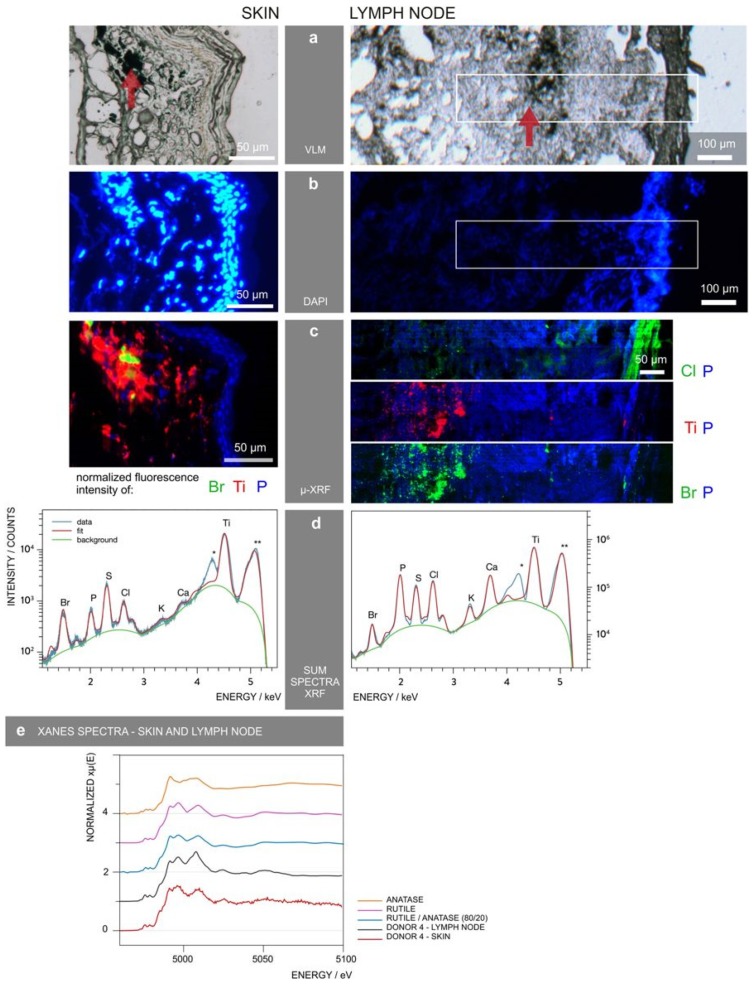
Synchrotron X-ray fluorescence was used to identify and locate tattoo particle elements in skin and lymph nodes of a donor. (**a**) Visible light microscopy (VLM) images of the area were mapped by μ-XRF and tattoo pigments were indicated by a red arrow. (**b**) 4′,6-diamidino-2-phenylindole (DAPI) staining of the tissues showing the cell nuclei. (**c**) μ-XRF maps of P, Ti, Cl, and/or Br. For the lymph node, these areas are marked in (**a**,**b**). (**d**) Average μ-XRF spectra over the full area displayed in (**c**) * diffraction peak from sample support; ** scatter peak of the incoming beam. (**e**) Ti K-edge micro-X-ray absorption near edge structure (μ-XANES) spectra of skin and lymph node compared to the spectra of rutile, anatase, and an 80/20 rutile/anatase mixture calculation. This figure was obtained from *Scientific Reports*, 11395, Copyright 2017, Springer Nature. This work was published under a CC BY 4.0 license (http://creativecommons.org/licenses/by/4.0/) [166].

TiO_2_ NPs may also influence bacteria in humans in another significant manner. Wu and Xing et al. investigated the impact of oral consumption of anatase and rutile NPs found as additives in sweets, on mice gut microbiota [159]. The treatment did not affect the microbiota diversity but shifted the amounts of each strain in a time-dependent manner, which could, to an extent, actually be due to different bacteria having a propensity for the material. The impact of rutile NPs was more pronounced than that of anatase NPs [159]. TiO_2_ NPs applied to human skin could effect skin microbiota (and even gut microbiota from ingestion) in an analogous manner. The symbiotic relationship of the human microbiome plays an extremely important function in helping to regulate the immune system [160,161]. While the cutaneous innate and adaptive immune response modulates the skin microbiota, the skin microbiota informs the immune system of the external environment and foreign bodies. Any change due to nanoparticle interaction with skin microbiota can disrupt the innate and adaptive immune response. Billions of T cells are found in the skin that are responsible for responding to pathogenic micro-organism. Animal studies have shown that TiO_2_ NPs (<100 nm) can translocate to the lymph nodes by lymphatic vessels and can activate dendritic cells [162], messenger cells between the innate and adaptive immune response These studies have also shown NP accumulation at hair follicles [162]. Hair follicles are now believed to help regulate the trafficking of immune cells [163,164]. Not much is understood about how TiO_2_ NPs affect the function of immune cells but it has been observed that the NPs can bind antigens and increase their persistance, possibly leading to an increased antigenicity [165]. This behavior could be the source of the small number of reported allergies toward Ti-containing implants [22,23]. A recent study evaluating the biological fate of TiO_2_ NPs in pigment used in tattooed human skin by synchrotron X-ray fluorescence (XRF) revealed that they translocate to lymph nodes (Figure 6) [166].

The possibility for a portion of TiO_2_ NPs to become solubilized exists because of the presence of hydroxyacids such as citrate, as previously discussed, that can induce solubilization by chelation [31,33,68,69,70,71,72,73,76]. In a related study, it has been shown that the Ti(IV) tricitrate complex can be photoreduced by UV, producing a Ti(III) species as confirmed by electron paramagnetic resonance [167]. The structure of this species has not been fully characterized, although Ti(III) citrate species are notoriously excellent reducing agents [168]. An anaerobic environment was used to generate the Ti(III) product in addition to several hours of UV irradiation but it is not known whether it could form under aerobic conditions. If Ti ions are generated within sunscreen, then it is likely to be of the Ti(IV) form. At present, we can only speculate on the movement of Ti ions into skin cells and their translocation into the body. The Fe(III)-binding transferrin family of proteins have been attributed to the binding and transport of Ti(IV) within the human body [31,32,33,73,169,170,171,172,173]. One other member of this family that may play a role is melanotransferrin (MTf). MTf exists mainly in a glycosylphosphatidylinositol-anchored membrane form predominantly in the epidermis of the skin, although a secreted form does exist [174,175,176]. Although the innate functions of MTf are not yet clear, it does appear to play a role in Fe(III) cellular uptake [176] and presumably should be able to do the same for Ti(IV) in skin cells. If solubilized Ti(IV) ions translocate with TiO_2_ NPs into the body and eventually the bloodstream, then citrate molecules and serum transferrin would be expected to capture this pool of Ti(IV) and regulate its blood speciation. Soluble Ti(IV) ions in human cells and tissue can demonstrate detrimental effects similar to TiO_2_ NPs and several that are distinct. Ti ions have been reported to induce substantial damage in macrophages by interrupting the cell division, oxidative stress, and other inflammatory reactions [177]. They can cause DNA structural modifications and result in DNA fragmentation [30] possibly by phosphate hydrolysis [170,178]. Piekoszewski et al. reviewed several of the potential problems that soluble Ti(IV) can cause in the body [29]. It is important to remember that several of these issues may be overcome by the synergistic regulation of Ti(IV) by citrate and sTf.

## 6. Conclusions

The diverse use of TiO_2_ NPs is increasing every day by a variety of industries around the world, especially in the food and cosmetic fields. The tremendous use of these materials poses health hazards but in our opinion, TiO_2_ NPs should be used strategically with great consideration for their formulation in sunscreens to avoid a detrimental effect on a wide range of living organisms and the environment at large. While these particles display a variety of bioactive properties that can be fine-tuned for beneficial human use and to clean up the environment, if not designed properly, they can undergo uncontrolled release and even solubilization that can lead to unpredictable speciation and ultimately devastating effects.
